# Incorporating Germanium Oxide into the Glass Phase of Novel Zinc/Magnesium-Based GPCs Designed for Bone Void Filling: Evaluating Their Physical and Mechanical Properties

**DOI:** 10.3390/jfb9030047

**Published:** 2018-07-31

**Authors:** Basel A. Khader, Omar Rodriguez, Mark R. Towler

**Affiliations:** 1 Department of Mechanical and Industrial Engineering, Ryerson University, Toronto, ON M5B 2K3, Canada; basel.khader@ryerson.ca (B.A.K.); omaralejandro.rodrig@ryerson.ca (O.R.); 2 Li Ka Shing Knowledge Institute, St. Michael’s Hospital, Toronto, ON M5B 1T8, Canada

**Keywords:** germanium oxide, bioactive glass, glass polyalkenoate cement, injectability, wettability

## Abstract

The structural role of Germanium (Ge), when substituting for Zinc (Zn) up to 8 mol % in the 0.48SiO_2_–0.12CaO–0.36ZnO–0.04MgO glass series, was investigated with respect to both the glass chemistry and also the properties of glass polyalkenoate cements (GPCs) manufactured from them. The Network connectivity (NC) of the glass was calculated to increase from 1.83 to 2.42 with the addition of GeO_2_ (0–8 mol %). Differential thermal analysis (DTA) results confirmed an increase in the glass transition temperature (*T_g_*) of the glass series with GeO_2_ content. X-ray photoelectron spectroscopy (XPS) showed an increase in the ratio of bridging oxygens (BO) to non-bridging oxygens (NBO) with the addition of GeO_2_, supporting the NC and DTA results. ^29^Si magic angle spinning nuclear magnetic resonance spectroscopy (^29^Si MAS-NMR) determined a chemical shift from −80.3 to −83.7 ppm as the GeO_2_ concentration increased. These ionomeric glasses were subsequently used as the basic components in a series of GPCs by mixing them with aqueous polyacrylic acid (PAA). The handling properties of the GPCs resulting were evaluated with respect to the increasing concentration of GeO_2_ in the glass phase. It was found that the working times of these GPCs increased from 3 to 15 min, while their setting times increased from 4 to 18 min, facilitating the injectability of the Zn/Mg-GPCs through a 16-gauge needle. These Ge-Zn/Mg-GPCs were found to be injectable up to 96% within 12 min. Zn/Mg-GPCs containing GeO_2_ show promise as injectable cements for use in bone void filling.

## 1. Introduction

Fractures of the wrist can be common at any age, but individuals suffering from osteoporosis are at a greater risk [1]. The most common wrist fractures are those of the distal radius and scaphoid. There has been a rise in both internal and external fixation of fractures regarding the distal radius and scaphoid as a result of increased patient expectation and the implementation of improved fixation techniques [2]. Although these techniques provide some stability for the fracture, there are disadvantages with metal-based fixation techniques, such as infection, pin loosening and potential for non or delayed unions [3]. Calcium phosphate cements (CPCs) (Norian SRS; Norian, Cupertino, California) and Cortoss (Stryker, MI, USA) have been used as bio-adhesives for treating wrist fractures. It has been reported in the literature that CPCs are biocompatible and osteoconductive; however, they often provide insufficient stability without the use of pins and k-wires [4]. Although Cortoss has been reported to offer sufficient strength and bonding to aid in the healing and reconstruction of the fractured bone, it has been found to cause exothermic reactions during setting, resulting in thermal necrosis of healthy bone tissue [5].

There is potential for Glass Polyalkenoate Cements (GPCs) to be used in void filling and fracture fixation because of their adhesive nature, their radio-opacity and their load-bearing ability. GPCs set by an acid-base reaction between an ionomeric glass and aqueous poly(acrylic acid) (PAA), and unlike Cortoss, GPCs have the ability to bond chemically with bone [6]. CPCs are also able to bond chemically to bone, but they are brittle in tension, restricting their use in load-bearing fracture fixation [4]. GPCs have been employed in both dental [7,8,9] and ear, nose and throat (ENT) applications [10,11], and efforts have been made to modify these materials for orthopedic applications. GPCs set with no significant heat evolution or shrinkage and possess mechanical properties similar to bone [12,13]. They bond chemically to hydroxyapatite (HA) and release beneficial ions that can help prevent caries, such as fluorine or silver [14,15]. The advantages of using GPC in dentistry include the GPC’s biocompatibility, bioactivity, high-dimensional stability, good resistance to cohesive failure, negligible shrinkage upon setting, among other advantages [16,17,18,19]. Numerous applications of GPCs have been investigated to expand its current use to other applications. For example, titanium (Ti) has been substituted for silicon (Si) in the glass phase of GPCs to improve the cement’s mechanical and biological properties [20]. Zinc (Zn) and silver (Ag) ions have been incorporated into the glass phase of GPCs because of their antimicrobial activity [21], and strontium (Sr) ions have been substituted for calcium (Ca) ions to increase radio-opacity of the cement and to stimulate bone formation around the implantation site [22,23,24]. Despite these amendments, the literature does not contain any reports of viable injectable GPCs for orthopedic applications as amendments to improve mechanical properties usually result in a deleterious effect on handling properties.

Germanium (Ge) Oxide is an inorganic compound [25] which takes the role of a network former when incorporated into an ionomer glass and is theoretically capable of isomorphically replacing Si in the network [26]. Ge had been previously incorporated into borate-based ionomer glasses (BGG) by Zhang et al. [27,28]. There was an increase in the setting and working time (handling properties) of the GPCs formulated from these glasses as a result of Ge incorporation, as this decreases the number of non-bridging oxygens (NBOs) in the glass network decreasing the working and setting times of the GPCs [29]. Dickey et al. [27,30,31] reported on Ge-based ionomeric glasses and reported that glass reactivity decreased with Ge, ZrO_2_ and Na_2_O incorporation, resulting in GPCs with extended working time (T_w_) of up to 10 min, long setting time (T_s_) up to 36 min, and compression strengths over 30 MPa after 30 days maturation [25]. Dickey et al. [27,30,31] failed to evaluate whether these cements were injectable or not for use in vertebroplasty fixation. The work herein expands partly on the current authors’ previous work on zinc (Zn) silicate-based GPCs by evaluating the effect of incrementally replacing the Zn in the glass component with Ge [25]. In the authors’ previous study, Zn-GPCs containing up to 6 mol % Ge with incorporated bovine serum albumin (BSA) exhibited decreased working times (T_w_) and setting times (T_s_), with increased compressive strengths after 30 days maturation [25]. The resultant Ge-containing Zn-GPCs, then, were injectable, but set too slowly for purpose [25].

The objective of this study was to investigate the incorporation of Ge into the glass phase of a novel Zn/Mg-GPC glass composition (0.48SiO_2_, 0.36ZnO, 0.12CaO, and 0.04MgO), particularly with respect to the influence that the glass changes had on the handling properties of Zn/Mg-GPCs manufactured from them.

## 2. Materials and Methods

### 2.1. Glass Synthesis

Three glasses were produced containing germanium oxide (GeO_2_) using the control glass TK10 (SiO_2_-CaO-ZnO-MgO) [32] while substituting zinc oxide (ZnO) with GeO_2_ up to 8 mol % (TK16, TK17 and TK18) as shown in Table 1. GPCs made with glasses in this series that contained less than 6.5 mol % GeO_2_ were not injectable. Appropriate amounts of analytical grade silicon oxide (SiO_2_), ZnO, calcium oxide (CaO), magnesium oxide (MgO) and GeO_2_ (Sigma Aldrich, Oakville, ON, Canada) were weighed out in a plastic tub and mixed by hand for 20 min using a spatula. The glass batches were then transferred to platinum (Pt) crucibles for firing (1480 °C, 1 h). The melts were subsequently shock quenched into water and the resulting frits were dried, ground using a ball mill (Retsch PM100 Planetary Ball Mill, Retsch GmbH, Haan, Germany) at 420 RPM for 15 min and sieved through a 45 μm mesh. The glass powders that passed through the sieve were subsequently used for characterization purposes and as reagents in a new series of GPCs named TK10, TK16, TK17 and TK18 after the respective parent glasses.

### 2.2. Structural Characterization of Glasses

#### 2.2.1. Network Connectivity

The network connectivity (NC) of the glasses was calculated using Equation (1), considering the molar compositions of the glasses (Table 1), where Si^4+^ and Ge^4+^ were considered network formers [10,32,33], each contributing four bridging oxygens (BO) to the glass network [34]. Zn^2+^ and Mg^2+^ behave as network intermediates and have previously been identified as acting primarily as network modifiers in similar glasses; each of these elements contribute 2 non-bridging oxygen (NBO) to the glass network [32,35].

(1) $$\mathrm{NC}=\frac{\mathrm{No}.\;\mathrm{BOs}-\mathrm{No}.\;\mathrm{NBOs}}{\mathrm{Total}\;\mathrm{No}.\;\mathrm{Bridging}\;\mathrm{Species}}$$

where, NC = Network Connectivity; BO = Bridging Oxygens; NBO = Non-Bridging Oxygens.

#### 2.2.2. X-Ray Diffraction (XRD)

Diffraction patterns were collected using an X’Pert PRO (PANanlytical Inc., St. Laurent, QC, Canada). The glass powder samples from the TK series were attached to a stainless-steel disc. These samples were scanned in the range 0° < 2θ < 100° at a scan step size 0.05 and a step time of 10 s. A generator voltage of 45 kV and a tube current of 40 mA were employed using a Cu *Kα* X-ray source.

#### 2.2.3. Particle Size Analysis (PSA)

PSA measurements were collected using a Coulter Ls 100 Fluid module Particle size analyser (Beckman Coulter, Fullerton, CA, USA). The Glass powder samples from the TK series were tested in the range of 2–60 μm with a run length of 60 s. Glycerol was used as the suspension fluid and was maintained at a temperature of 23 °C. The average diameters (*n* = 5) were recorded for the cumulative volume distribution at 10% (d_10_), 50% (d_50_) and 90% (d_90_).

#### 2.2.4. Scanning Electron Microscopy and Energy Dispersive X-ray Analysis (SEM-EDX)

A JEOL Co. JSM-6380LV (JEOL Ltd., Tokyo, Japan) SEM was used to obtain backscattered electron (BSE) images on glass particles. An EDX Genesis Energy-Dispersive Spectrometer (JEOL Co. JSM-6380LV, JEOL Ltd., Tokyo, Japan) was used to study the composition of the glass particle distribution. All EDX spectra were collected at 20 kV.

#### 2.2.5. Differential Thermal Analysis (DTA)

A combined differential thermal analyser–thermal gravimetric analyser (DTA–TG; SDT 2960 Simultaneous DSC-TGA, TA Instruments, New Castle, DE, USA) was used to measure the thermal properties for each glass sample, i.e., glass transition (*T_g_*), crystallization (*T_c_*) and melting (*T_m_*) temperatures. A heating rate of 20 °C/min was employed using an air atmosphere with alumina in a matched platinum crucible as a reference. Sample measurements were performed every 6 s between 30 °C and 1200 °C. Data analysis was conducted using NETZSCH Proteus software, V. 6 (Netzsch–Gerätebau GmbH, Selb, Germany), with *T_g_* taken at the onset temperature where the slope of the heat curve changes, *T_c_* taken at the peak of an exothermic reaction, and T_m_ taken at the peak of an endothermic reaction.

#### 2.2.6. X-ray Photoelectron Spectroscopy (XPS)

The as-fabricated glasses were tested on a ThermoFisher Scientific K-Alpha XPS spectrometer (Thermo Fisher Scientific, E. Grinstead, UK). Survey spectra were collected at low energy resolution (pass energy, 200 eV). High resolution scans (pass energy 50 eV, step-size 0.1 eV) were obtained for the spectral regions of interest and each element associated with the TK glass series. A monochromatic Al Kα X-ray source was used, with the 400 μm spot size. Charge compensation was provided utilizing the combined e−/Ar+ flood gun and the position of the energy scale was adjusted to place the main C1s feature (C-C adventitious C) at 284.6 eV. The instrument and all data processing were performed using the Avantage v5.926 software (ThermoFisher Scientific, Waltham, MA, USA) and the sensitivity factors (modified Scofield factors [36]) provided with the instrument.

#### 2.2.7. Magic-Angle Spinning-Nuclear Magnetic Resonance Spectroscopy (MAS-NMR)

^29^Si MAS-NMR spectrum for each glass was recorded at 8T on a Unity Inova 300 FT-NMR spectrometer (Varian, Palo Alto, CA, USA), equipped with a cross polarization-magic angle spinning (CP-MAS) probe. The glass samples were placed in a zirconia sample tube with a diameter of 7 mm. The sample spinning speed at the magic angle was 5 kHz. ^29^Si MAS NMR spectra were taken at 59.59 MHz with 7.0-ls pulse length (pulse angle, p/2), 100-s recycle delays, where the signals from 2126, 1837 and 1880 pulses were accumulated for TK10, TK16, TK17 and TK18, respectively. The chemical shifts for all glass samples were reported in ppm. The external reference used to measure the chemical shifts was polydimethylsilane (−34 ppm vs. TMS 0 ppm).

### 2.3. Cement Preparation

The powdered poly(acrylic acid) (PAA) with a molecular weight of 50,000, coded PAA35 with a maximum particle size of <45 μm was provided by Advanced Healthcare Limited (Tonbridge, Kent, UK). The cement samples were prepared by mixing 1 g of glass powder with 0.360 g of PAA35 powder and 0.640 mL double deionized (DDI) water on a glass plate. This produced cements with Powder: Liquid (P:L) ratios of 1:1 for all the GPC series.

### 2.4. Determination of Working and Setting Times

Working time (T_w_) of each cement (*n* = 5) was measured in ambient air using a stopwatch and was defined as the period of time from the start of mixing during which it was possible to manipulate the material without having an adverse effect on its properties [37,38]. Setting times (T_s_) of the cement samples were measured according to ISO9917 [38]. A mould with internal cross-sectional dimensions of 10 × 8 mm^2^ was placed on an aluminum foil and filled with the mixed cement; 60 s after mixing the cement samples, it was placed on a metal block with dimensions of 8 × 75 × 100 mm^3^ and kept in an oven maintained at 37 °C for 30 s. A Vicat needle indenter (mass 400 g) was lowered onto the surface of the cement 90 s after mixing the cement. The needle was kept on the surface for 5 s, the indent produced was then observed. This was repeated every 30 s until the needle was unsuccessful at producing a complete circular indent when viewed at ×2 magnification. The net T_s_ (*n* = 5) was recorded for each GPC composition.

### 2.5. Preparation of Injectable GPCs (iGPCs)

**Figure 1:**
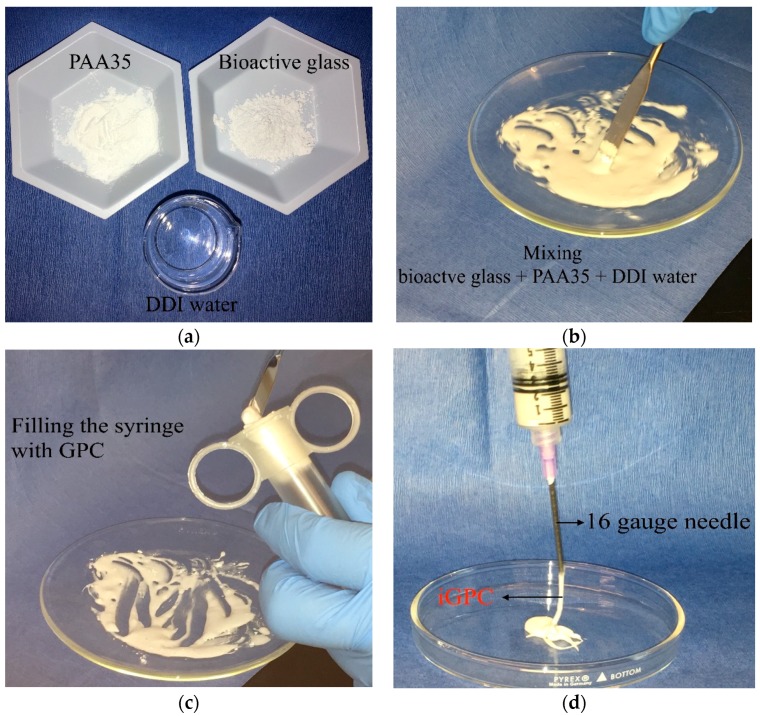
The procedure for the injection technique of the Zn/Mg based GPCs containing Ge. (**a**) shows the cement components prior to mixing; (**b**) shows manual mixing with a spatula; (**c**) shows the transferring of the cement to the syringe prior to dispensing; (**d**) shows the iGPC being dispensed.

Injectability was evaluated using disposable 10 mL syringes with a 16-gauge needle that extruded the GPCs through the syringe using hand pressure (Figure 1). Each individual syringe was filled with 3 g of mixed cement after 30 s of mixing using a spatula; the cement from the first syringe was extruded as soon as it was filled, the cement from the second syringe was extruded one min after the filling time; this process continued for the remaining syringes in one min increments until hand pressure was unable to inject any further cement. This procedure was repeated (*n* = 5) for each iGPC. The weight of the injected cement was then measured and injectability for that time was calculated using the following Equation (2) [39]:

(2) $$\mathrm{Injectability}\;\left(\%\right)=\frac{\left(\mathrm{Cement}\;\mathrm{weight}\;\mathrm{expelled}\;\mathrm{from}\;\mathrm{the}\;\mathrm{syringe}\right)}{\left(\mathrm{total}\;\mathrm{cement}\;\mathrm{weight}\;\mathrm{before}\;\mathrm{injection}\right)}100\%$$

### 2.6. Evaluation of Mechanical Properties

#### 2.6.1. Determination of Compressive Strength

Compressive strengths $\left(\sigma_{C}\right)$ of each cement sample were evaluated in accordance with ISO9917 [38]. An STM United Tensile Tester (United Testing Systems, Inc., Huntington Beach, CA, USA) fitted with a ±2 kN load cell at a crosshead speed of 1 mm min^−1^ was used to test the cement samples (*n* = 5). Moulds (4 mm Ø × 6 mm height) according to ISO9917 [38], were filled using a disposable 10 mL syringe with a 16-gauge needle (injection technique) to excess with freshly mixed cement then covered with acetate sheet [32]. After 1 h of incubation at 37 °C the samples were de-moulded and then incubated in DDI water (37 °C) for 1, 7 and 30 days. $\sigma_{C}$ was calculated according to Equation (3) [38]:

(3) $$\sigma_{C}=\frac{4\rho }{\pi d^{2}}$$

where ρ = maximum applied load (N); *d* = diameter of sample (mm).

#### 2.6.2. Determination of Biaxial Flexural Strength ($\sigma_{f}$)

The biaxial flexural strength *(*$\sigma_{f}$*)* of the cements samples (*n* = 5) were evaluated with the same method used by Williams et al. [40]. Cements disc samples were tested after being incubated in 10 mL DDI water for 1, 7, and 30 days. Testing was performed on an STM United Tensile Tester (United Testing Systems, Inc., Huntington Beach, CA, USA) using a load cell of ±2 kN at a crosshead speed of 1 mm min^−1^. $\sigma_{f}$ was calculated according to Equation (4) [40].

(4) $$\;\sigma_{f}=\frac{\rho }{t^{2}}\left|0.63\;\ln\left(\frac{r}{t}\right)+1.156\right|$$

where ρ = fracture load (N), t = sample thickness (mm), r = radius of the support diameter (mm).

### 2.7. Determination of pH and Ion Release

#### 2.7.1. Ion Release Profile

The concentrations of Si, Ca, Zn, Mg and Ge ions released from the injectable GPCs for 1, 7 and 30 days were determined by analysing the water extracts in which samples of each set of iGPCs were stored using a Perkin Elmer Atomic Absorption Spectrometer 800 (AAS800, Perkin Elmer, Waltham, MA, USA). Samples (*n* = 5) of each injectable cement (8 mm Ø, 2 mm thick) were then stored for 1, 7 and 30 days in 10 mL aliquots of DDI water kept at 37 °C in lidded containers. Following removal of cement samples from their aliquots, a 1:10 dilution of the storage water was made using DDI water. Calibration standards for Si, Ca, Zn, Mg and Ge elements were prepared from a stock solution on a gravimetric basis. Five target calibration standards were prepared for each ion with 1, 3, 5, 7 and 10 parts per million (ppm) concentrations with DDI water was used as a blank. Samples for Ca, Zn, Mg and Ge ion analysis were diluted in a ratio of 1:10; that is, each 1 mL of concentrated sample was mixed with 10 mL of DDI water while samples for Si analysis were diluted in a ratio of 1:30. A pilot study was conducted to determine the appropriate ratio for dilution of all elements.

#### 2.7.2. pH Analysis

iGPC samples (6 mm high and 4 mm diameter) were prepared from each glass type for pH testing. Sample solutions were prepared by exposing iGPC samples (*n* = 5) in 10 mL of DDI water and incubated (37 °C) for 1, 7, 30 days. Changes in the pH of the DDI water were monitored using a Corning 430 pH meter. Prior to testing, the pH meter was calibrated using pH buffer solution 4.00 ± 0.02 and 7.00 ± 0.02 (Fisher Scientific, Pittsburgh, PA, USA).

### 2.8. Determination of Micro-CT Imaging

*Micro-CT* was examined according to the ASTM F640 “Standard Test Methods for Determining Radiopacity for Medical Use” which was applied for monitoring the position of permanently implanted medical devices [41]. In order to ensure that the adhesives are radiologically detectable to facilitate long term monitoring [42], radio-opacity was determined and compared to a cortical bone standard using a SCANCO Medical AG Micro-CT40 scanner (Fabrikweg, Brüttisellen, Switzerland). An initial scan was performed, giving overall X-ray and 3D images from which an area of focus was then selected for scanning at full resolution (40 μm). Each of the TK injectable GPC samples were paired with a hydroxyapatite (HA) calibration standard. Images were then reconstructed and the density of the cement samples measured using SCANCO Medical’s software (SCANCO Medical, Bruttiselen, Switzerland). Radio-opacities were calculated as “mineral per volume” in the CT scan and can be considered a bone mineral density and was expressed as mass per unit volume (g/mL), where HA calibration phantom was included with the system as a standard.

### 2.9. Contact Angle Measurements (Wettability)

iGPC discs have been prepared using the same method as the $\sigma_{f}$ samples (Section 2.6.2) for contact angle measurements. Each sample was placed in DDI water at 37 °C for 1, 7 and 30 days. Measurements were taken using a Theta Lite Optical Tensiometer (Biolin Scientific, Espoo, Finland). DDI water was used to perform the contact angle analysis. Contact angle data were recorded in ambient air (23 ± 1 °C) using a static sessile drop method, measuring 10 s post drop placement. The DDI water drops (6 μL) were deposited with a micro-syringe (Biolin Scientific) on each iGPC samples (*n* = 5) that had been previously matured for 1, 7 and 30 days.

### 2.10. Statistical Analysis

One-way analysis of variance (ANOVA) was used to analyze the data for handling and mechanical properties using non-parametric Kruskal–wallis. A Post hoc Bonferroni test was used to compare the relative means and to report the statistically significant differences when *p* ≤ 0.05. Statistical analysis was performed using SPSS software (IBM SPSS statistics 21, IBM Corp., Armonk, NY, USA).

## 3. Results

The addition of Ge to the control glass TK10 (NC = 1.83) in amounts of up to 8 mol % resulted in a network connectivity increase in glasses TK16 (NC = 2.33), TK17 (NC = 2.36) and TK18 (NC = 2.42).

### 3.1. X-ray Diffraction (XRD)

**Figure 2:**
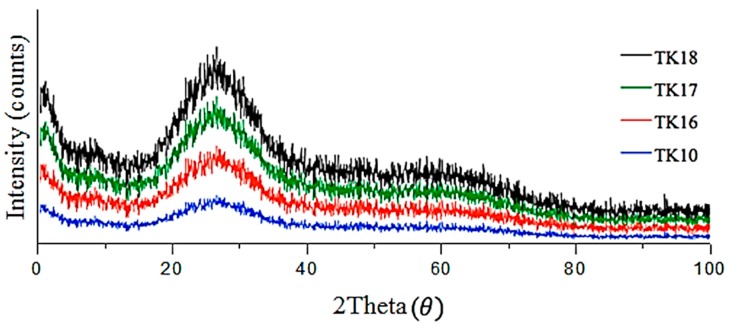
XRD patterns of the formulated glasses (TK10, TK16-TK18) series confirming each glass is fully amorphous.

XRD patterns were recorded for each of the as-fired glasses and are depicted in Figure 2. XRD confirmed that all fired glasses were fully amorphous; no crystalline species were detected in TK10, TK16-TK18.

### 3.2. Particle Size Analysis (PSA)

All glasses evaluated were found to have similar particle size (Table 2) with a mean particle size of around 6 μm.

### 3.3. Scanning Electron Microscopy and Energy Dispersive X-ray Analysis (SEM-EDX)

**Figure 3:**
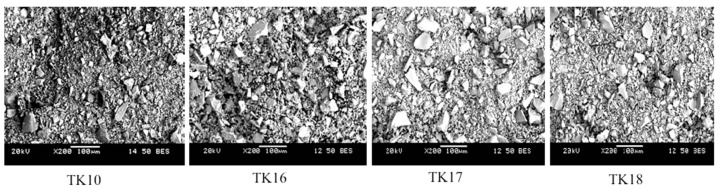
SEM micrograph images showing the glass particles of the glass series.

In Figure 3, SEM images for each glass are shown, with image contract resulting from the different atomic number of the glasses’ compositional elements.

EDX was performed during scanning electron microscopy to confirm that the elemental contents incorporated in the starting mixtures for glass firing were present in comparable amounts in the glasses (Table 3) formulated from them, also confirming a reduction in Zn and an increase in Ge.

### 3.4. Differential Thermal Analysis (DTA)

DTA results (Table 4) were used to determine the *T_g_*, *T_c_* and *T_m_* of each glass. *T_g_* did not differ significantly between TK10, TK16 and TK17, however, a significant difference was found between TK10 and TK18. *T_c_* showed a significant difference between all components of the glass series, with the exception of TK16 to TK17. *T_m_* found no significant difference with addition of GeO_2_ up to 6.5 mol %; yet, a significant difference was present when incorporating more than 6.5 mol % of GeO_2_.

### 3.5. X-ray Photoelectron Spectroscopy (XPS)

**Figure 4:**
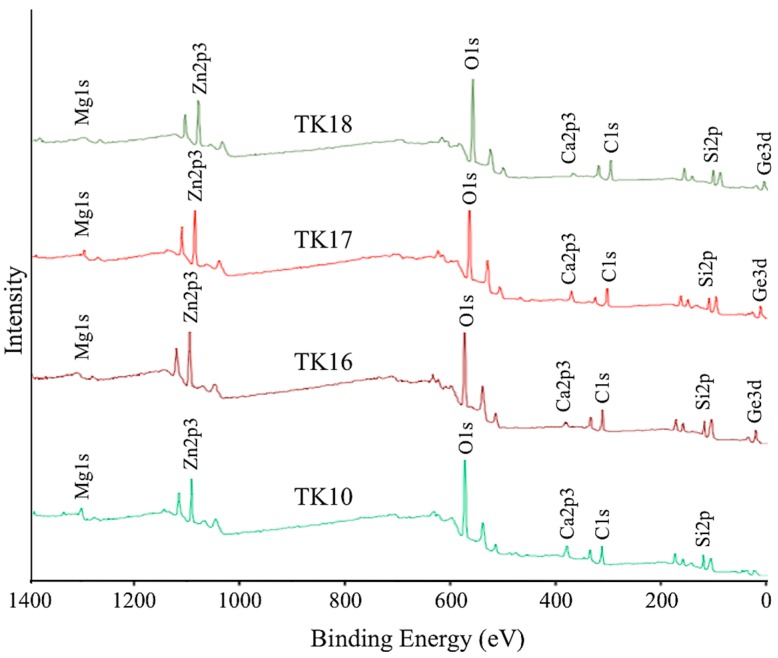
Wide scan XPS spectra from the surface of the glass series being investigated.

XPS survey scans of TK10, TK16, TK17 and TK18 are presented in Figure 4 and confirm the starting formulation of the glasses that contain Mg1s, Zn2p3, O1s, Ca2p3, Ge3d and Si2p. The carbon (C1s) peak was detected in the survey scans and was used as a reference point. Elemental compositions of O1s, Si2p, Zn2p3, Ca2p3, Mg1s and Ge3d are shown in Table 5.

Table 6 displays the peak for Ge, which was shown at 0 eV (TK10), 32.17 eV (TK16), 32.21 eV (TK17) and 32.21 eV (TK18). High resolution XPS spectra were also performed on Calcium (Ca2p), Silica (Si2p), Magnesium (Mg1s) and Zinc (Zn2p), and peak positions (eV) are also depicted in Table 6. No significant shift was found with the addition of Ge through the glass series.

**Figure 5:**
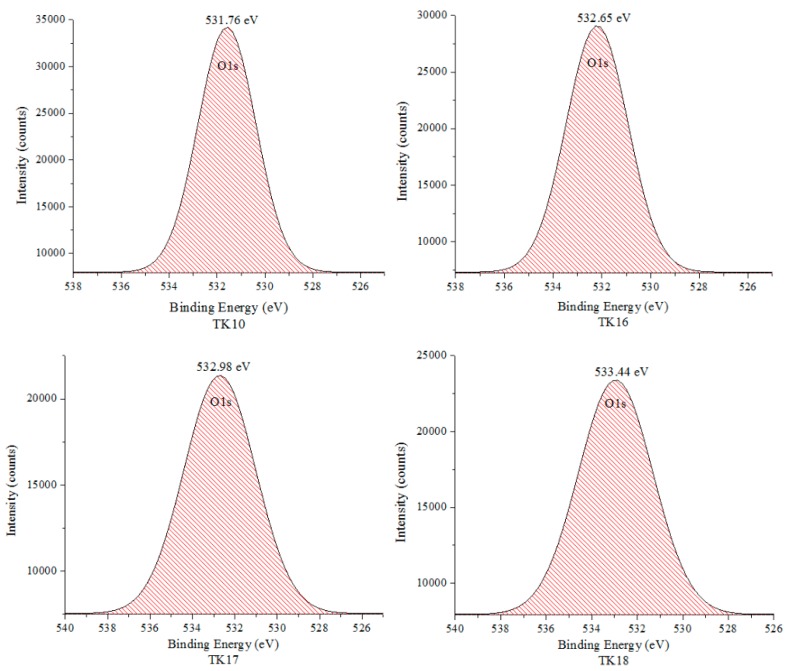
Curve fitting of the O1s high resolution core level spectra for the glass series.

Figure 5 clearly represents the curve fitting for Oxygen (O1s) obtained from Origin (OriginLab, Northampton, MA, USA); the binding energies (B.E.) of TK10, TK16, TK17 and TK18 were found to shift slightly from 531.76 to 533.44 eV while increasing the concentration of Ge.

**Figure 6:**
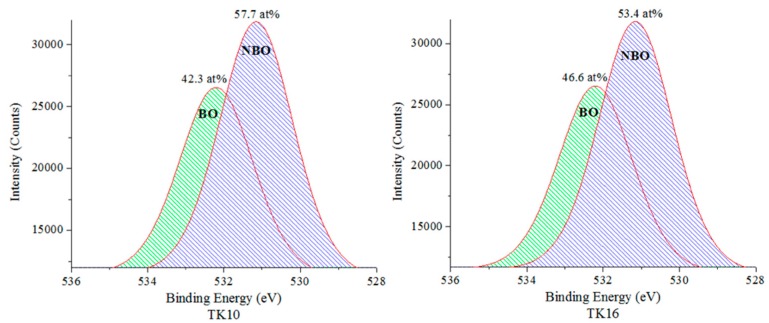

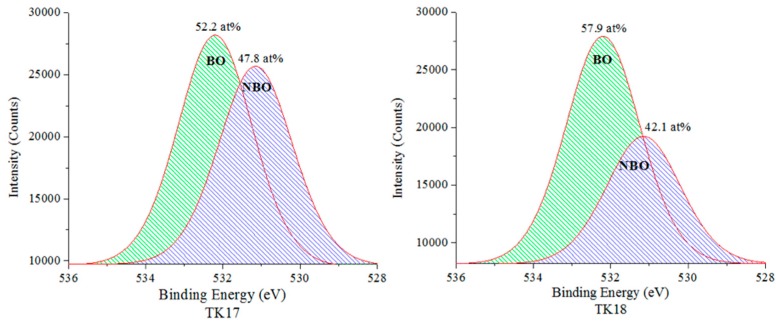
Curve fitting for the BO and NBO of the TK glass series with respect to O1s.

Figure 6 and Table 7 show the peak positions for the BO and the NBO and their corresponding at %. Figure 6 was obtained by deconvoluting the curves from Figure 5 into the NBO and BO using Origin (OriginLab, Northampton, MA, USA). BO and NBO stayed at 531.6 eV and 532.8 eV regardless of GeO_2_ content. However, an increase was found in the BO content at the expense of the NBO content due to an increase of the GeO_2_ content up to 8 mol %, thus increasing BO:NBO ratio.

### 3.6. ^29^Si Magic-Angle Spinning-Nuclear Magnetic Resonance Spectroscopy (MAS-NMR)

**Figure 7:**
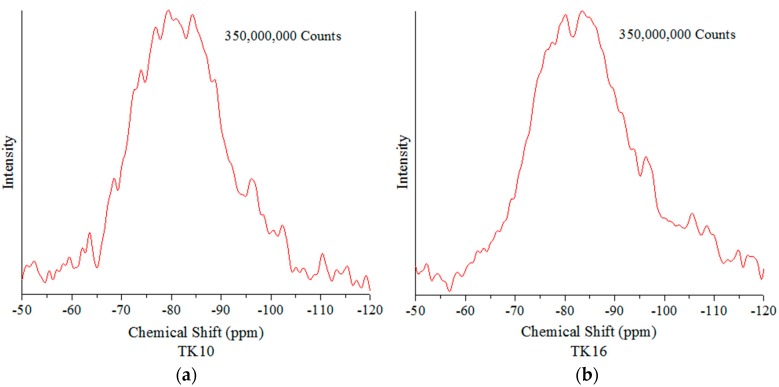

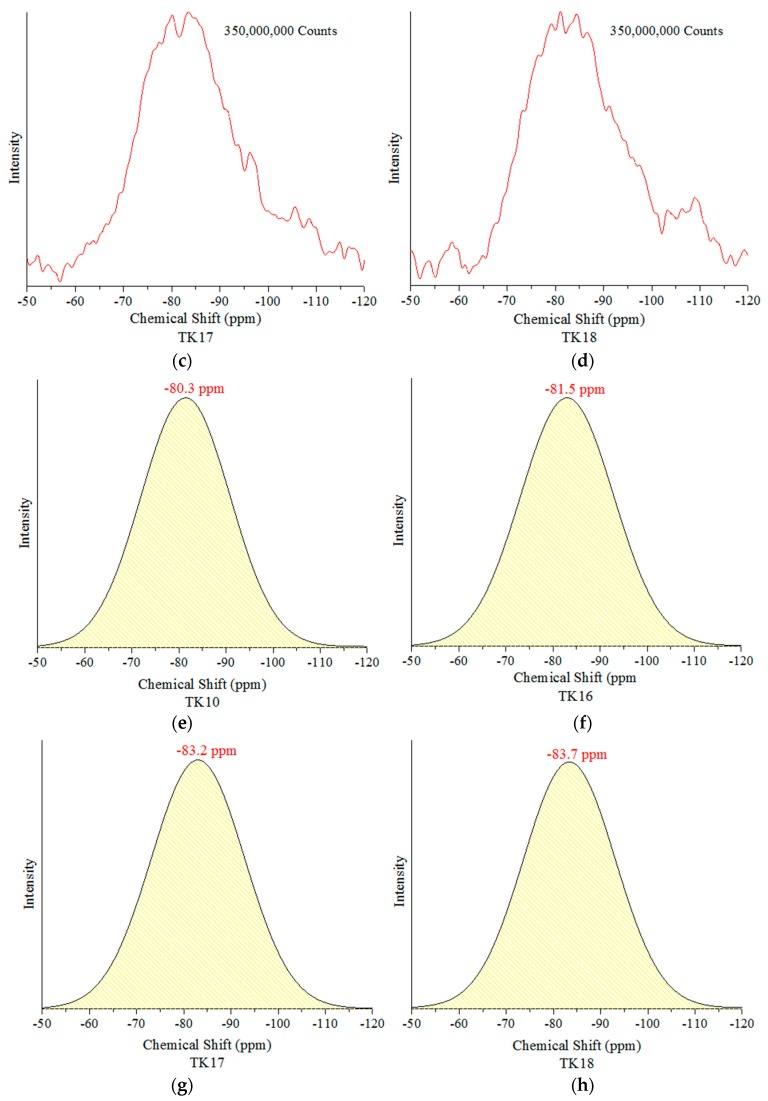
Curve fitting of the ^29^Si NMR spectra: (**a**) expanded spectrum of TK10; (**b**) expanded spectrum of TK16; (**c**) expanded spectrum of TK17; (**d**) expanded spectrum of TK18; (**e**) simulated (curve fitted) spectrum of (**a**); (**f**) simulated (curve fitted) spectrum of (**b**); (**g**) simulated (curve fitted) spectrum of (**c**); (**h**) simulated (curve fitted) spectrum of (**d**).

Chemical shifts in ^29^Si MAS-NMR represent structural changes around the Si atom, which lie in the region of −100 to −60 ppm for SiO_4_ tetrahedra [43]. Figure 7 shows the ^29^Si MAS-NMR spectra of the glass series (TK10, TK16, TK17 and TK18). The ^29^Si MAS-NMR glass samples showed broad resonances between −70 ppm to −100 ppm. It can be seen that there are differences with the chemical shift of TK10 (−80.3), TK16 (−81.5), TK17 (−83.2) and TK18 (−83.7) when increasing the amount of GeO_2_. Figure 7a–d is the expanded versions of the NMR spectra of the glass series. Figure 7e–h is the corresponding curve fitted (simulated) spectra.

### 3.7. The Influence of GeO Incorporation on Working and Setting Times of GPCs

**Figure 8:**
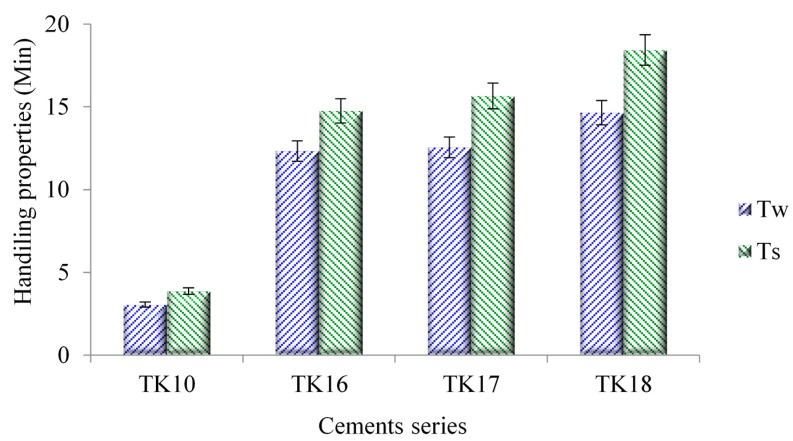
T_w_ and T_s_ for Ge-containing Zn-GPCs. Error bars represent standard deviation from the mean (*n* = 5).

The T_w_ and T_s_ of the cement series were evaluated with respect to the increasing concentration of GeO in the glass phase, and are presented in Figure 8, each data point being the average of five tests. T_w_ were recorded and found to be 3, 12, 13 and 15 min for TK10, TK16, TK17 and TK18, respectively. T_s_ were recorded and found to be 4, 15, 16 and 18 min for TK10, TK16, TK17 and TK18, respectively.

### 3.8. Injectability of TK GPCs

**Figure 9:**
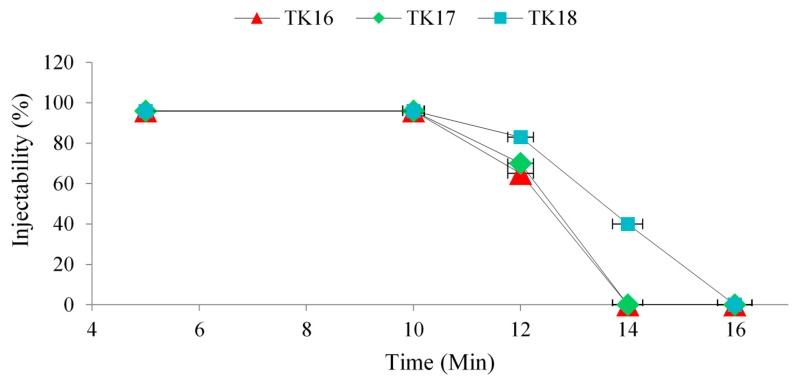
Injectability of iGPCs containing Ge depending on the time, (*n* = 5) for each cement.

The injectability of the GPCs was evaluated using Equation (2). TK10 was not injectable and was removed from further investigation. TK16, TK17 and TK18 iGPC maintained 96% injectability after 10 min (once the 3 mL syringe had extruded all the cement, ~4% of the cement remained in the 16 gauge needle), but the iGPCs were no longer injectable after 14 min for TK16 and TK17, while TK18 was no longer injectable after 16 min, as shown in Figure 9 and Table 8.

### 3.9. Evaluation of Mechanical Properties of Compressive Strength and Biaxial Flexural Strength

**Figure 10:**
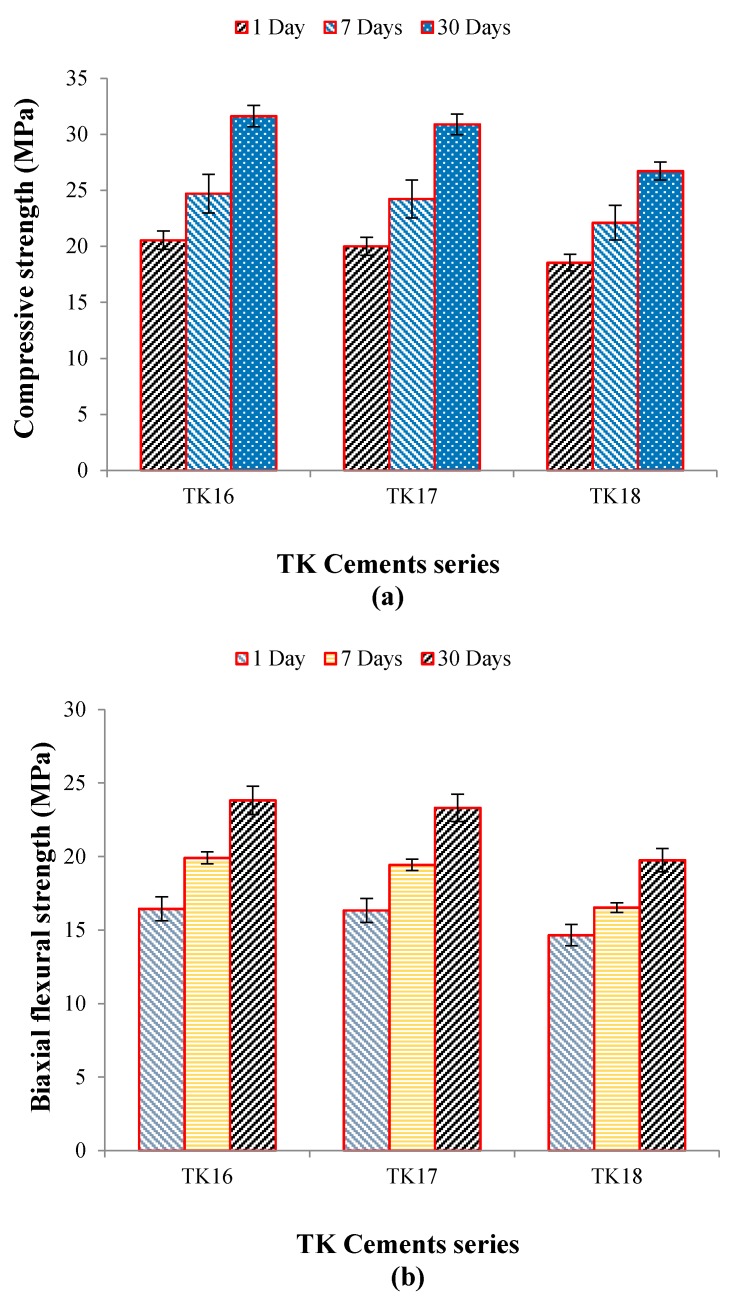
$\sigma_{C}$ (**a**) and $\sigma_{f}$ (**b**) strengths of the iGPC series when aged in DDI water for 1, 7 and 30 days. Error bars represent standard deviation from the mean (*n* = 5).

$\sigma_{C}$ and $\sigma_{f}$ of the iGPCs tested over 1, 7 and 30 days are presented in Figure 10a,b, respectively. The highest $\sigma_{C}$ and $\sigma_{f}$ were found to be 32 MPa and 24 MPa, respectively, and were obtained for TK16 after 30 days maturation.

### 3.10. Determination of Ion Release and pH

#### 3.10.1. Ion Release Profile

**Figure 11:**
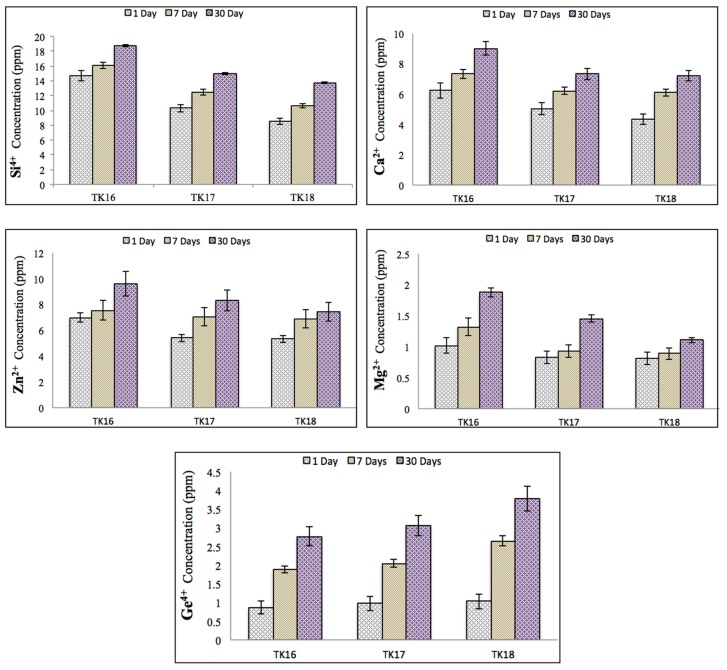
Ion release profiles of Si^4+^, Ca^2+^, Zn^2+^, Mg^2+^ and Ge^4+^ ions during iGPCs ageing in DDI water. Error bars represent standard deviation from the mean (*n* = 5).

Ion release from the iGPCs were measured cumulatively over 1, 7 and 30 days and are tabulated in Figure 11. As expected, ion release (Si, Ca, Zn, Mg and Ge) increased with maturation. However, the incorporation of Ge into the glass resulted in lower ion release for the Si, Ca, Zn and Mg ions at the same time point, with the obvious exception of Ge, which, understandably, increases in line with its content in the precursor glass from which it elutes.

#### 3.10.2. pH Analysis

**Figure 12:**
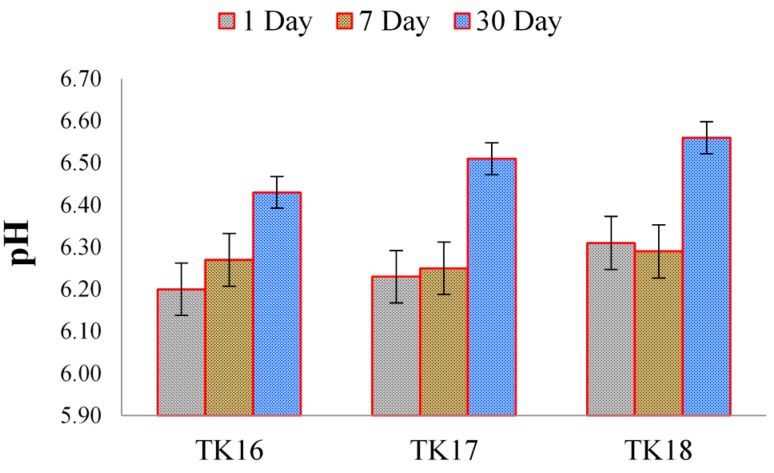
pH measurements during iGPCs ageing in DDI water for 1, 7 and 30 days. Error bars represent standard deviation from the mean (*n* = 5).

Changes in the pH of DDI water (pH = 6.00) initially in the presence of TK injectable cements were evaluated, with results shown in Figure 12, respectively. After 30-days incubation, no significant difference (*p* < 0.05) was observed as a function of the amount of GeO_2_ incorporated for the cement series up to 8 mol %, with the TK cements averaging a pH of 6.34.

### 3.11. Determination of Micro-CT Analysis

**Figure 13:**
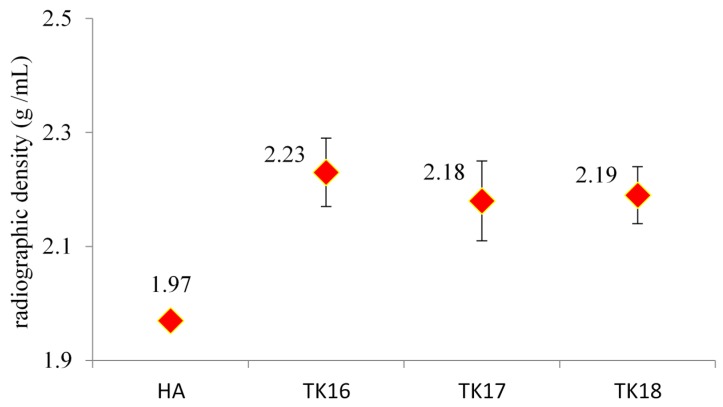
The density of the iGPC samples (TK16, TK17 and TK18) recorded using units g/cm^3^. Error bars represent standard deviation from the mean (*n* = 3).

The results of the *Micro-CT* testing are shown in Figure 13. Figure 13 shows the 3D radiographic images and X-ray scans of the iGPC samples. The cement densities were higher (2.19 to 2.23 gHA/mL) than that of the HA standard, the density of which was measured as 1.97 gHA/mL (Figure 13)

### 3.12. Contact Angle Measurements (Wettability)

**Figure 14:**
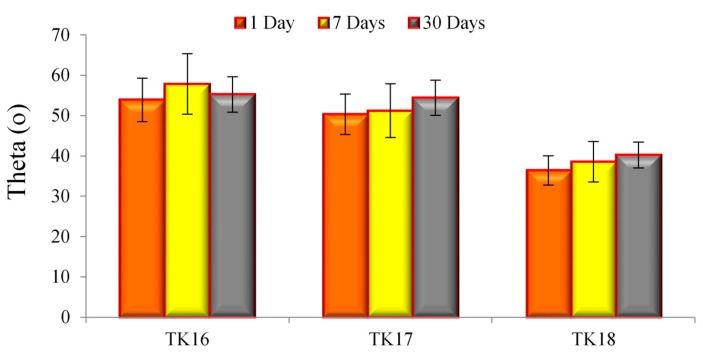
Contact angle measurements for the iGPC samples over 1, 7 and 30 days. Error bars represent standard deviation from the mean (*n* = 5).

Figure 14 shows the contact angle measurements of DDI water over the GPC surfaces tested over 1, 7 and 30 days. The contact angle showed no significant difference over 1, 7 and 30 days for all iGPCs. However, it can also be seen that the increase of Ge in the glass composition appears to reduce the contact angle of the cement. TK16 was found to have the highest contact angle over 1 (~54°), 7 (~58°) and 30 (~55°) days in comparison to TK17 and TK18. No statistical difference was found between TK16 and TK17 for 7 days (*p* = 0.062) and 30 days (*p* = 0.083). Yet, a statistical difference was found between TK16 and TK18 for 1 (*p* = 0.001), 7 (*p* = 0.008) and 30 (*p* = 0.008) days.

## 4. Discussion

### Structural and Thermal Characterization of the Glass Series

XRD confirmed that no crystalline species were present in all the glass series after being fired. Therefore, it can be assumed that any change in the properties of the glasses would not be related to phase changes or separation in the glasses.

The particle size of the glass phase will impact the rheological properties of GPCs formulated from them; for example, increases in the surface area of the glass component will increase T_w_ and reduce T_s_ [35]. However, all glasses had similar particle sizes (around 6 µm), and so it was fair to conclude that any measurable changes in handling properties of GPCs made from these glasses would be related to the chemistry, rather than physicality, of the glass phase.

Differential thermal analysis (DTA) was used to detect any changes in the *T_g_* as a result of the incorporation of Ge (Table 4). In this instance a shift in *T_g_* can indicate that structural changes are occurring within the glass as the concentration of Ge was increased. *T_g_* was found to increase between TK10 and TK18 as the concentration of Ge was increased in the glass melt from 0 mol % (TK10) to 8 mol % (TK18). This shift suggests increased glass stability, which may be attributed to the formation of BO groups, which likely exists as Si–O–Ge groups are formed in the glass, agreeing with the network connectivity calculation. As expected the increase of BO requires increased energy to melt the glass [32], therefore, *T_m_* increased from 1055 °C to 1139 °C with increasing Ge content up to 8 mol %. This was assumed to be due to the increasing ratio of BO:NBO when increasing the concentration of a former element (Ge).

XPS shows a shift of O1s that could be due to increased Ge concentration, in turn allowing for an increase in the at % of BO (Figure 7). A decrease in the at % of O1s was found (15.6 at %) in the NBO content in the glass with the addition of 8 mol % Ge in the glass phase. As expected, Ge increases when substituted for Zn, the network connectivity as compared to the proportion acting as a former across the glass series increases. This agrees with previous work by the authors [25], who found that the network connectivity increases while increasing the Ge concentration. The significant change in the BO:NBO ratio (Figure 6) with the addition of 8 mol % of Ge was in agreement with the increased NC and the increased *T_g_* between TK10 and TK18 where Ge addition to the glass was a network former. The O1s peaks are broad with a full width at half maximum (FWHM) of peaks at ~1.9 eV suggesting a multi local environment of the oxygen atoms in terms of BO and NBO species. NBO groups (Si–O–NBO) are known to disrupt the glass network by depolymerizing the Si–O–Si bonds [39]. This was regarded as a positive attribute as this facilitates the ion exchange process, which in turn increases bioactivity of these materials [44,45]. There is a constant interchange of ions among the set cement and the nearby body fluid or hard tissue [46], the exchange of ions with surrounding tissues at the implant site is the basis of the osteo-conductive and bone-bonding properties associated with GPCs [47]. Regarding this work, the O1s of the TK glass series containing Ge shifted to a higher BE which was indicative of increasing the BO content in the glass, therefore, expected to increase the handling properties of the iGPCs and allowing it to be an injectable cement [25].

^29^Si MAS-NMR determined a chemical shift in ppm in a negative direction, as presented with TK16, TK17 and TK18, and was indicative of an increase in BO species attached to the Si within the glass [25], aligning with the XPS results presented earlier in this manuscript (Section 3.5). When BO content increases the peak will shift in a negative direction, and when NBO content increases the peak will shift in a positive direction [25,48,49]. It was believed that Si resonances (−86 ppm) are linked with Q^3^ species, while resonances that occur at −78 ppm are linked with Q^2^ structures [50]. It has been stated in the literature that ^29^Si resonances ranging from −60 and −83 ppm are linked with the Q^0/1^ structural unit and that Q^2^ structures are accepted to resonate at −78.9 ppm [50]. Replacing Zn^2+^ with Ge^4+^ in the glass network has an effect on the glass structure, when replacing Ge^2+^ for Zn^2+^ there are slight chemical shift differences as shown in the Figure 8.

Handling properties of the GPCs show a statistically significant difference (*p* <0.05) with Ge incorporation from TK10 to TK18. The increasing trends experienced in both T_w_ (~3–15 min) and T_s_ (~4–18 min) resulted from the introduction of Ge to the glass phase, which may result in a decreased susceptibility to acid attack, the glasses releasing more cations into the environment, increasing carboxylic (COO-) metal bonding rates [6]. The introduction of Ge ions, which have a 4+ charge, may also increase the bonding extend of the unbonded COO- molecular chains [45]. NC increases with Ge content. However, decreasing ZnO content reduces preferential acid attacks on ZnO_4_ sites, which lead to an increase in T_w_ and T_s_, regardless of the increase in BOs, as confirmed by XPS [51]. The difference in injectability between the three GPCs was due to the increase in Ge concentration. It was found that T_w_ and T_s_ increased in TK16, TK17 and TK18 with the addition of Ge as previously explained. TK10 was found to be uninjectable due to its short T_w_ and T_s_. Thus, TK10 has been removed from further experiments in this paper.

Si/Zn-iGPCs (SiO_2_-CaO-ZnO-Na_2_O-GeO_2_) previously reported by the authors exhibited $\sigma_{C}\;$ of ~37 MPa at 30 days maturation and T_s_ ~ 38 min [25], comparable to the cements reported here which had $\sigma_{C}$ of ~33 MPa with shorter T_s_ ~13 min. TK16 with 6.5 mol % GeO_2_ was found to have a longer setting time compared to TK10 with 0 mol % GeO_2_ and a shorter T_s_ than the TK17 (7 mol % GeO_2_) and TK18 (8 mol % GeO_2_) (Figure 11). This may be due to TK16 having a higher percentage of NBOs compared to TK17 and TK18, which results in a greater cross-linking of the PAA and increased $\sigma_{f}$ of the iGPCs [32]. No significant difference (*p* > *0.05*) was found in $\sigma_{C}$ and $\sigma_{f}$ between TK16 and TK17 cements. However, a significant decrease (*p =* 0.008) was found in $\sigma_{C}$ between TK16 and TK18 and a significant decrease (*p* = 0.032) was also found between TK17 and TK18 with respect to maturation after 30 days. There was a significant decrease (*p* = 0.000) found in $\sigma_{f}$ between TK16 and TK18 and a significant decrease (*p* = 0.008) was also found between TK17 and TK18 with respect to maturation after 30 days. Additionally, XPS results confirmed that a higher concentration of NBOs compared to BOs were present in the TK16 glass, as opposed to TK17 and TK18 glasses, increasing degradability of the glass network compared to the other glasses, therefore decreasing the mechanical properties [30,32].

The release of Si^4+^, Ca^2+^, Zn^2+^ and Mg^2+^ decreased with GeO_2_ content (Figure 12) and was also dependent on immersion time. This was expected as GeO_2_ was substituted with ZnO in TK16, TK17 and TK18 up to 8 mol %, therefore, increasing the NC and allowing more ions to bond to the network of the glass phase to retard cement degradation. Figure 12 (Ge) represents the release of Ge^4+^ ions which was found to increase in TK16, TK17 and TK18 with increasing concentrations of Ge, peaking at ~2.8 ppm (TK16), ~3.1 ppm (TK17), ~3.8 ppm (TK18) after 30 days of immersion. Again, this is attributed to the longer setting reaction of cements with increased Ge content, which retards initial cross-linking after the attack of the PAA on the glass structure. This phenomenon can also be attributed to the slow reaction of the Ge^4+^ ions with the carboxylic groups.

*Micro-CT* has confirmed that iGPCs are radiologically suited for long term monitoring. There were no changes found in the density of the iGPC series with the incorporation of GeO_2_.

Small contact angles (<90°) correspond to high wettability [51]. Contact angle measurements were found to decrease with Ge incorporation, therefore, confirming the results of the setting reaction of the iGPCs in which the quicker set has a higher contact angle, and therefore, surface energy. Generally, all cement samples were found to be favorable to water (hydrophilic), it is assumed that the cell tissue of the bone has the ability to be seeded onto the surface of the iGPCs [52].

## 5. Conclusions

Al-free GPC compositions reported in the literature were not clinically useful as they failed to achieve an appropriate balance between handling and mechanical properties. In this study, we have shown that the addition of GeO_2_ at up to 8 mol % to a Zn/Mg-containing, Aluminium-free ionomer glass increased the BO in the glass network and resulted in iGPCs formulated from these glasses with extended T_w_ and T_s_. Thus, these materials were injectable up to 96% within 12 min of mixing, with mechanical properties exceeding 31 MPa in compression ($\sigma_{C}$) and 23 MPa in flexure ($\sigma_{f}$) after 30 days maturation. Incorporating Ge into the glass phase of these GPCs resulted in rheology, hydrophilicity, strength, radio-opacity and injectability suitable for wrist fracture fixation.

## Figures and Tables

**Table 1:** Glass formulations (mol %).

Glass Formulations	SiO_2_	CaO	ZnO	MgO	GeO_2_
TK10	48	12	*36*	4	-
TK16	48	12	*29.5*	4	*6.5*
TK17	48	12	*29*	4	*7*
TK18	48	12	*28*	4	*8*

**Table 2:** Particle size analysis data for the glass series.

Glass	Mean (µm)	d_10_ (µm)	d_50_ (µm)	d_90_ (µm)	S.D. (µm)
TK10	5.9	2.5	4.4	11.4	1.12
TK16	5.7	2.4	4.5	11.1	1.31
TK17	6.1	2.3	4.2	11.9	1.43
TK18	6.0	2.4	4.6	11.5	1.11

**Table 3:** Composition in mol % as verified by EDX.

Elements	TK10 (σ)	TK16 (σ)	TK17 (σ)	TK18 (σ)
O	33.2 (0.2)	33.1 (0.1)	34.5 (0.1)	32.4 (0.1)
Si	36.7 (0.2)	34.6 (0.2)	36.5 (0.1)	36.3 (0.2)
Ca	6.8 (0.1)	5.4 (0.2)	3.1 (0.3)	4.7 (0.2)
Zn	20.2 (0.2)	18.4 (0.3)	17.6 (0.1)	17.8 (0.3)
Mg	3.1 (0.1)	3.8 (0.3)	3.1 (0.2)	2.6 (0.1)
Ge	--	4.7 (0.1)	5.2 (0.1)	6.2 (0.2)

(σ): Standard deviation.

**Table 4:** Thermal events in the glass series (*n* = 1) as a function of GeO_2_ content incorporated at the expense of ZnO.

Thermal Event	TK10	TK16	TK17	TK18
*T_g_* (°C)	687	691	696	698
*T_c_* (°C)	809	823	831	847
*T_m_* (°C)	1055	1048	1073	1139

**Table 5:** Actual glass compositions (mol %) as determined by XPS survey scans.

Peaks	TK10	TK16	TK17	TK18
O1s	41.1	42.4	44.3	44.9
Si2p	29.1	27.3	27.2	27.1
Zn2p3	20.3	18.7	18.2	15.2
Ca2p3	5.3	4.7	3.8	3.2
Mg1s	3.2	3.7	3.4	3.3
Ge3d	-	4.2	5.1	6.3

**Table 6:** Peaks positions (eV) for the core levels Si2p, Zn2p3, Ca2p3, Mg1s and Ge3d obtained from high resolution XPS spectra.

XPS Peak	TK10	TK16	TK17	TK18
O1s	531.76	532.65	532.98	533.44
Si2p	102.16	102.32	102.33	102.31
Zn2p3	1022.52	1022.48	1022.48	1022.41
Ca2p3	347.54	347.53	347.51	347.52
Mg1s	1305.78	1305.78	1305.78	1305.78
Ge3d	-	32.17	32.21	32.21

**Table 7:** Peak positions (eV) for the BO and NBO peaks and their corresponding at %, obtained from the curve fitting of the O1s peaks, of the glass series.

O Peak	TK10	TK16	TK17	TK18
O1s (BO)	532.8	532.9	533.4	533.7
at %	42.3	46.6	52.2	57.9
O1s (NBO)	531.6	531.4	531.7	532.2
at %	57.7	53.4	47.8	42.1

**Table 8:** Cements series injectability % with respect to time (Min).

Cements Series	Time (Min)				
	5	10	12	14	16
TK16	96	96	65	0	0
TK17	96	96	70	0	0
TK18	96	96	83	40	0
